# Assessment of genetic diversity in *Trigonella foenum-graecum *and *Trigonella caerulea *using ISSR and RAPD markers

**DOI:** 10.1186/1471-2229-4-13

**Published:** 2004-07-30

**Authors:** Rakhee S Dangi, Meena D Lagu, Lal B Choudhary, Prabhakar K Ranjekar, Vidya S Gupta

**Affiliations:** 1 Plant Molecular Biology Group, Division of Biochemical Sciences, National Chemical Laboratory, Pune 411008. Maharashtra State. India; 2 Taxonomy and Biodiversity Division, National Botanical Research Institute, India. Lucknow 226001

## Abstract

**Background:**

Various species of genus *Trigonella *are important from medical and culinary aspect. Among these, *Trigonella foenum-graecum *is commonly grown as a vegetable. This anti-diabetic herb can lower blood glucose and cholesterol levels. Another species, *Trigonella caerulea *is used as food in the form of young seedlings. This herb is also used in cheese making. However, little is known about the genetic variation present in these species. In this report we describe the use of ISSR and RAPD markers to study genetic diversity in both, *Trigonella foenum-graecum *and *Trigonella caerulea*.

**Results:**

Seventeen accessions of *Trigonella foenum-graecum *and nine accessions of *Trigonella caerulea *representing various countries were analyzed using ISSR and RAPD markers. Genetic diversity parameters (average number of alleles per polymorphic locus, percent polymorphism, average heterozygosity and marker index) were calculated for ISSR, RAPD and ISSR+RAPD approaches in both the species. Dendrograms were constructed using UPGMA algorithm based on the similarity index values for both *Trigonella foenum-graecum *and *Trigonella caerulea. *The UPGMA analysis showed that plants from different geographical regions were distributed in different groups in both the species. In *Trigonella foenum-graecum *accessions from Pakistan and Afghanistan were grouped together in one cluster but accessions from India and Nepal were grouped together in another cluster. However, in both the species accessions from Turkey did not group together and fell in different clusters.

**Conclusions:**

Based on genetic similarity indices, higher diversity was observed in *Trigonella caerulea *as compared to *Trigonella foenum-graecum*. The genetic similarity matrices generated by ISSR and RAPD markers in both species were highly correlated (r = 0.78 at p = 0.001 for *Trigonella foenum-graecum *and r = 0.98 at p = 0.001 for *Trigonella caerulea*) indicating congruence between these two systems. Implications of these observations in the analysis of genetic diversity and in supporting the possible Center of Origin and/or Diversity for *Trigonella *are discussed.

## Background

The family Fabaceae includes many crops useful for food, forage, fiber, wood and ornamental purposes. In this family, a few legumes such as chickpea, soybean, fababean, fenugreek, lentil, pea etc. are consumed as grain legumes. The grain legumes are plants used as food in the form of unripe pods, mature seeds or immature dry seeds, directly or indirectly [1]. The grain legumes not only provide variety to human diet but they also supply dietary proteins for vegetarian populations that lack animal and fish protein in their diet. Considering the growing problem of malnutrition, use of legume species as high-protein food is very important. Moreover, legumes are also capable of symbiotic nitrogen fixation enriching the soil condition suitable for a crop following the legume crop [2].

The genus *Trigonella *is one of the largest genera of the tribe Trifoliatae in the family Fabaceae and sub-family Papilionaceae [3]. Among *Trigonella *species, *Trigonella foenum-graecum *(commonly known as fenugreek) is a flowering annual, with autogamous white flowers occasionally visited by insects. Indigenous to countries on the eastern shores of Mediterranean, fenugreek is widely cultivated in India, Egypt, Ethiopia, Morocco and occasionally in England [4]. *Trigonella foenum-graecum *is extensively grown in the tropical and subtropical regions of India. Different parts of the plant such as leaves and seeds are consumed in India. It is also used for medicinal purpose. According to ancient medicinal system, the Ayurveda, it is a herbal drugs that is bitter or pungent in taste. It is effective against anorexia and is a gastric stimulant [5]. Fenugreek is becoming popular around the world with its extract used to flavor cheese in Switzerland, artificial maple syrup and bitter-run in Germany, roasted seeds as coffee-substitute in Africa, seed powder mixed with flour as fortification to make flat-bread in Egypt, as an anti-diabetic herb in Israel, whole seed and dried plant used as insect and pest repellent in grain storage, and oil used in perfumery in France [6]. Research reports in recent years have indicated that fenugreek can be a remedy to diabetes by lowering blood sugar and cholesterol levels [7].

*T. caerulae*, from the same family and commonly known as the Blue fenugreek, on the other hand, is a less commonly grown herb. This flowering annual with autogamous blue flower is found in Alps and in the mountains of eastern and south eastern Europe. Terminal leaves are mainly used for cooking while young seedlings are eaten with oil and salt. Dried and powdered leaves as well as flowers are used for flavoring and coloring bread, cheese, etc in China and Germany. They are also used as a condiment in soups and potato dishes and a decoction of leaves is used as aromatic tea [8].

Grain legumes like *T. foenum-graecum *and *T. caerulea *although important in food and medicine are rarely grown outside their native habitat. Across the world only known and well-defined cultivars are grown in specific areas. Gene banks also harbor scanty germplasm collection of *Trigonella *species [9]. The neglected and the under-use status of these locally important crops indicates a risk of disappearance of important plant material developed over thousands of years of cultivation. One of the important factors restricting their large-scale production and development of better varieties is that very little information is available about their genetic diversity, inter and intraspecific variability and genetic relationship among these species. Therefore, attempts to analyze possible untapped genetic diversity become extremely essential for breeding and crop improvement. The present study was undertaken with the objective of analyzing genetic diversity in various accessions of *T. foenum-graecum *and *T. caerulea *representing various countries where they are grown using molecular marker technology.

## Results

### Assessment of genetic diversity in *T. foenum-graecum *and *T. caerulea *using ISSR and RAPD markers

A set of 100 ISSR primers was used for initial screening of 7 accessions of *T. foenum-graecum *of which 40 gave amplification. However, only 14 ISSR primers detected intraspecific variation in I7 accessions of *T. foenum-graecum *generating clear reproducible patterns and revealing 100 bands in the range of 500 bp to 2 kb. Among these seventy-two bands were polymorphic amounting to 72% polymorphism [Table 3]. Furthermore, during ISSR analysis 11 unique bands were obtained, where 6 were contributed by accession TMP = 8714 from Yemen, 4 by accession TMP = 8691 from Turkey, and 1 by accession TMP = 8685 from Iran. Similarly, in *T. caerulea*, of the 100 ISSR primers used for initial screening, 47 gave amplification. Of these, 18 primers detected intraspecific variation in 9 accessions of *T. caerulea *showing 93.64% polymorphism [Table 4]. With these 18 primers, 16 unique bands were produced, where 9 and 7 bands were contributed by accessions 206901 and 206486, respectively, from Turkey alone.

In case of RAPD analysis, 100 RAPD primers were used for initial screening in *T. foenum-graecum *of which 22 primers generated polymorphic patterns revealing 70.12% polymorphism [Table 3]. Eight unique bands were produced by these primers of which 3 were contributed by accession TMP = 8714 from Yemen, 2 by accession TMP = 8698 from Egypt, 1 by accession TMP = 8707 from Afghanistan and 1 each by accession TMP = 8691 and TMP = 8690 from Turkey. Similarly in *T. caerulea *of the 40 primers used for initial screening, 10 primers produced polymorphic pattern giving 95.83% polymorphism [Table 4]. Eight unique bands were produced with these primers wherein; the maximum numbers of unique bands (4 each) were again produced by the same accessions 206901 and 206486 from Turkey.

### Dendrogram analysis for *T. foenum-graecum *and *T. caerulea*

Genetic similarity was calculated from the Nei's similarity index value for all the 17 accessions of *T. foenum-graecum *considering ISSR and RAPD approaches individually as well as together. Based on ISSR markers alone, the similarity index values ranged from 0.69 to 0.92. These values were used to construct a dendrogram using Unweighted Pair Group Method with Arithmetic averages (UPGMA). In the ISSR based dendrogram *T. foenum-graecum *genotypes formed 4 clusters [Figure not shown]. The first cluster grouped together accessions from Afghanistan (8707), Canada (1065), Pakistan (8717,8718), Iran (8675), and Turkey (8690). The second cluster contained accessions from India (8686,8689,8675). Remaining one accession from India (8687) and one from Nepal (8706) formed the third cluster. The fourth cluster contained accessions from Egypt (8698,8679) and Turkey (8692). Accessions from Ethiopia (8696), Turkey (8691) and Yemen (8714) out grouped from the main clusters. Three accessions from Turkey analyzed in the present study fell into different clusters.

Based on RAPD markers alone, the similarity index values ranged from 0.71 to 0.91. In the RAPD based dendrogram, *T. foenum-graecum *genotypes formed 2 main clusters [Figure not shown]. The first cluster had two subgroups, the first subgroup contained accessions from Afghanistan (8707), India (8686,8687,8675), Turkey (8690) and Egypt (8698) while the second subgroup contained accessions from Pakistan (8717,8718), Turkey (8692), and Egypt (8679). The accession from Canada associated with these clusters. The second cluster contained accession from Iran (8685) and Nepal (8706) and the accession from India associated with this cluster. Accessions from Ethiopia (8696), Turkey (8691) and Yemen (8714) out grouped from these two clusters. In the RAPD based dendrogram also accessions from Turkey fell into different clusters.

**Figure 1:**
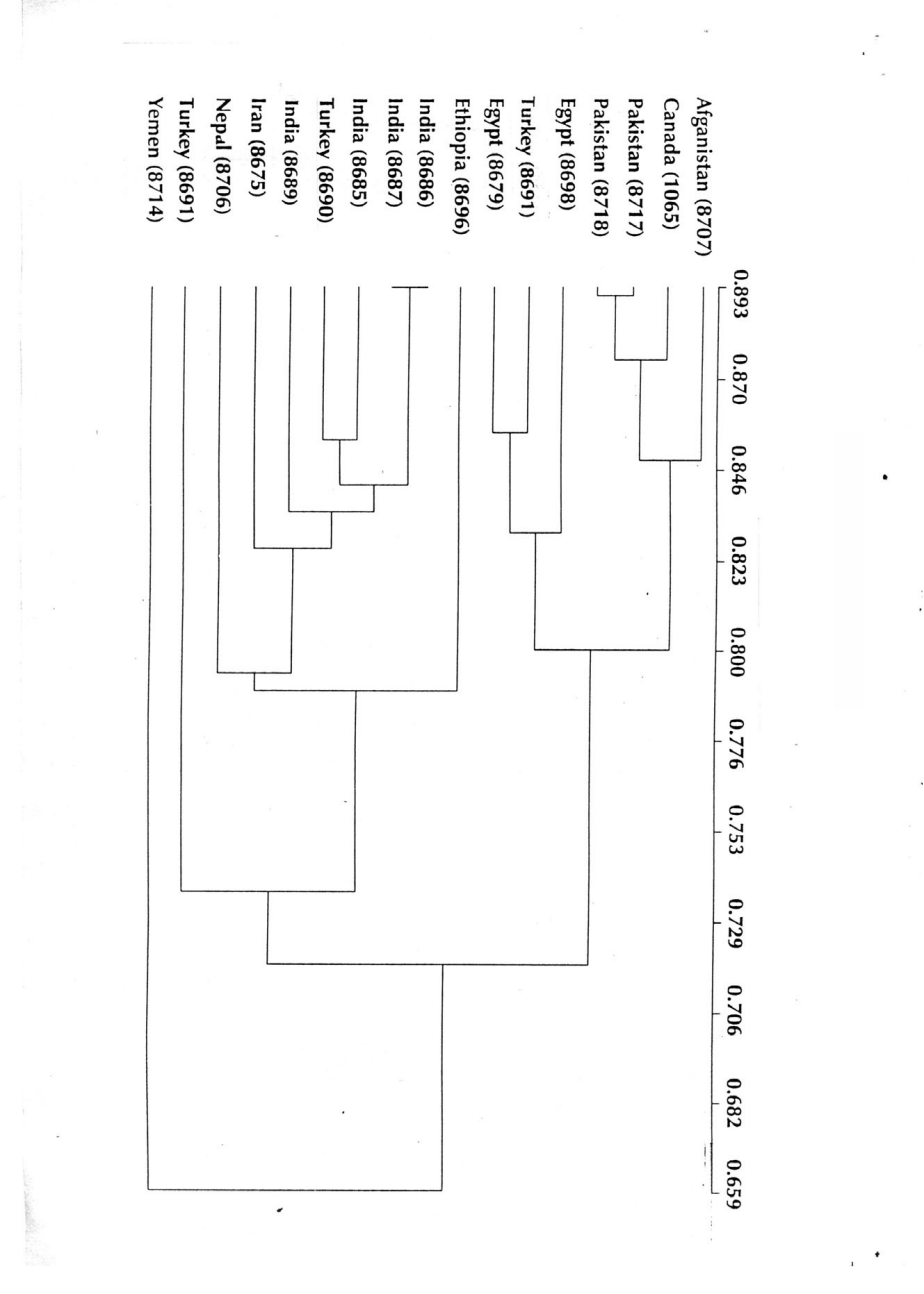
ISSR+RAPD dendrogram based on algorithm of Unweighted Pair Group Method With Arithmetic Averages in *T. foenum-graecum*.

Based on both the marker systems together the similarity index values ranged from 0.65 to 0.89 [Fig. 1]. Here the *T. foenum-graecum *accessions from Egypt (8698,8679) were grouped together. Accessions from Pakistan (8717, 8718) and Afghanistan (8707) were grouped together in one cluster. Accessions from India (8686,8689,8675,8687), Nepal (8706) and Iran (8685) were grouped together. However, all the three accessions from Turkey fell in different clusters and did not group among themselves. Bootstrapping was done using the WinBoot program to estimate the relative support at clades. The robustness of the cluster was not very strong in *T. foenum-graecum *(50–70%).

**Figure 2:**
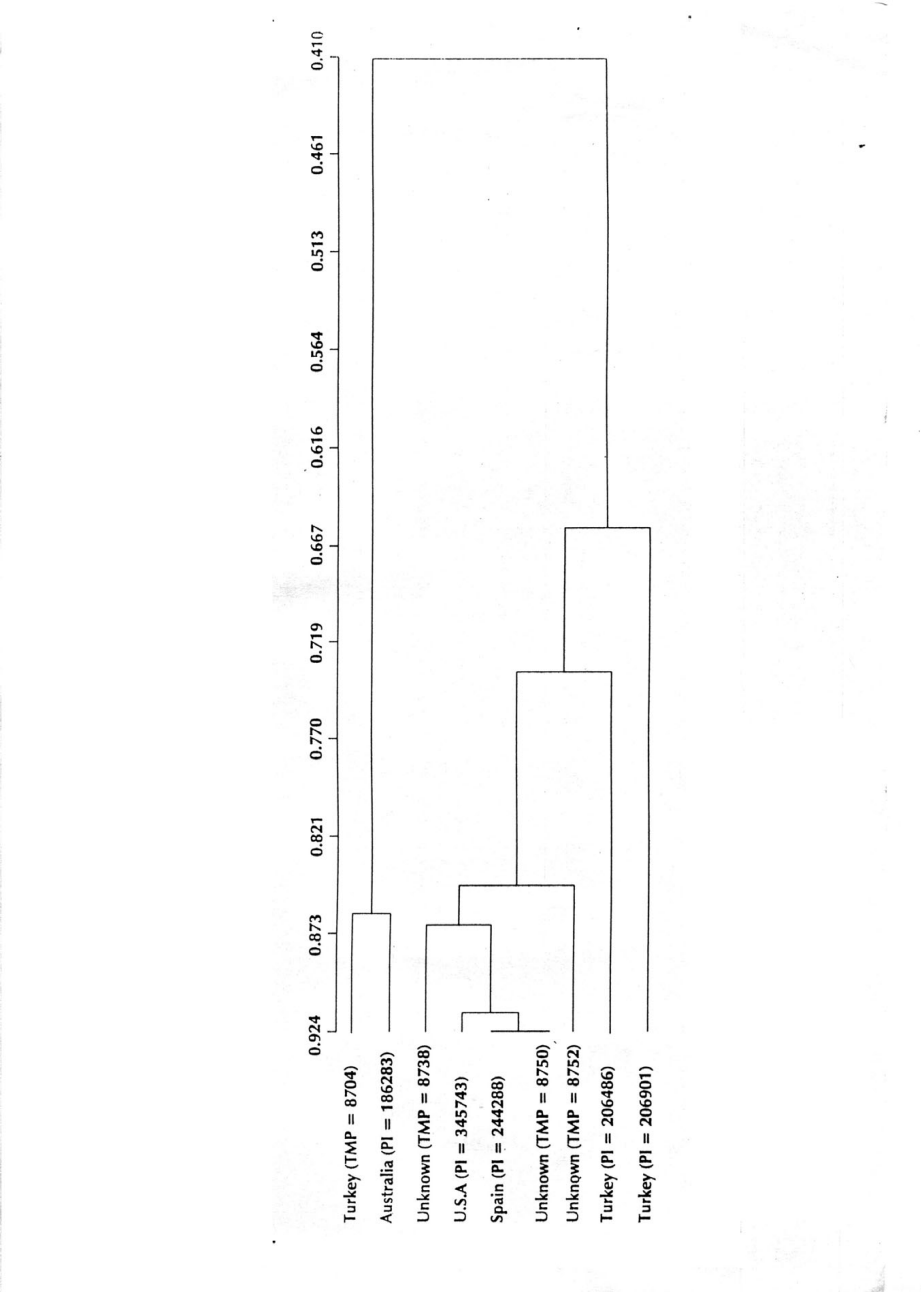
ISSR+RAPD dendrogram based on algorithm of Unweighted Pair Group Method With Arithmetic Averages in *T. caerulea*.

In *T. caerulea*, genetic similarity was calculated from the Nei's similarity index value considering ISSR and RAPD approaches individually as well as together. Based on ISSR marker system, the similarity index values ranged from 0.41 to 0.92 while that on the basis of RAPD markers ranged from 0.34 to 0.93. Similarity indices values based on both the marker systems together ranged from 0.38 to 0.92 indicating more diversity in case of *T. caerulea*. The dendrograms based on ISSR and RAPD markers showed similar clustering pattern when used individually [Figures not shown] as well as together [Fig. 2]. Here the dendrogram showed two main clusters. The first cluster grouped together accessions form Turkey (TMP = 8704) and Australia (PI = 186283). The second cluster contained 3 accessions of unknown geographic origin and 1 accession each from U.S.A (PI = 345743) and Spain (PI = 244288). The remaining 2 accessions i.e. accessions PI = 206486 and PI = 206901 from Turkey, out grouped from these two main clusters. Thus the accessions from Turkey did not cluster together as observed in *T. foenum-graecum*. The bootstrap values obtained using the WinBoot program in *T. caerulea *were very high (90–100%).

### Heterozygosity and marker index

Heterozygosity was calculated using ISSR and RAPD marker systems individually as well as together as detailed in Table 3 for *T. foenum-graecum *and in Table 4 for *T. caerulea*. In *T. foenum-graecum *the H_av _values calculated for ISSR, RAPD and ISSR+RAPD were 0.214, 0.203 and 0.211, respectively. In case of *T. caerulea *the H_av _values for the same were 0.330, 0.346 and 0.338, respectively. For both, *T. foenum-graecum *and *T. caerulea*, the H_av _values for ISSR and RAPD systems did not differ significantly. The marker index (MI) values calculated for ISSR and RAPD for *T. foenum-graecum *were 1.53 and 1.51, respectively while in *T. caerulea *they were 3.17 and 3.35, respectively. Thus MI values in both the species did not differ significantly for ISSR and RAPD systems.

### Correlation between measures of similarity

**Figure 3:**
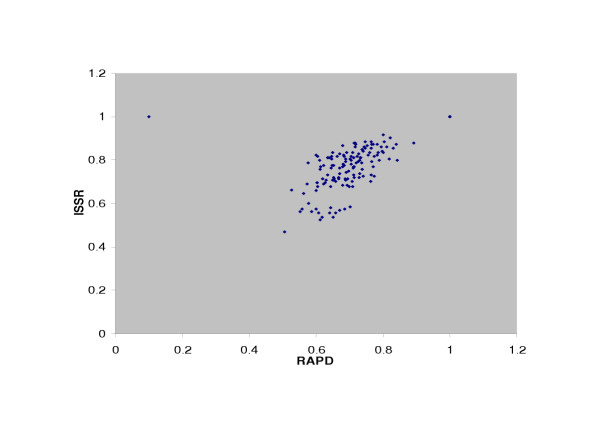
Regression analysis of similarity matrices generated using RAPD and ISSR marker systems in *T. foenum-graecum*.

**Figure 4:**
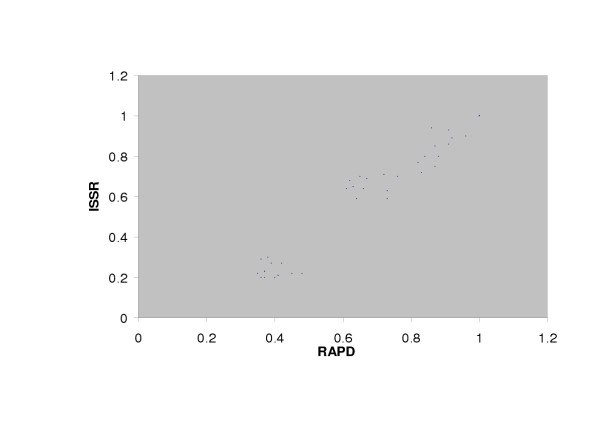
Regression analysis of similarity matrices generated using RAPD and ISSR marker systems in *T. caerulea*.

In *T. foenum-graecum*, when the similarity matrices generated using ISSR and RAPD markers were compared, a value of r = 0.78, at P = 0.001 indicated a good correlation between data generated by both the systems [Fig. 3]. Similarly when the similarity matrices generated using ISSR and RAPD systems were compared in case of *T. caerulea*, a value of r = 0.98 indicated a very good correlation between the two marker systems [Fig. 4].

## Discussion

The two marker systems, ISSR and RAPD used in the present study have also been used as effective tools to evaluate genetic diversity and to throw light on the phylogenetic relationships in *Brassica napi *[rapeseed, [10]], *Allium *sect. *Sacculiferum *[Alliaceae, [11]] and *Asimina triloba *[pawpaw, [12] and [13]]. Genetic diversity analysis using ISSR and RAPD markers in legumes like *Cicer *[[14] and [15]] and *Cajanus *[[17] and [18]] has been carried out in our own laboratory. These studies have given important clues in understanding species relationship, which may further assist in developing and planning breeding strategies. However, no such reports on genetic diversity using molecular markers were available in the genus *Trigonella*. In the present study, an attempt has been made to examine the level of genetic variation within *T. foenum-graecum *and *T. caerulea *accessions obtained from germplasm collection centers at Saskatoon (Plant Gene Resources of Canada) and Pullman (USDA-ARS Plant Introduction Station, Washington). The *T. foenum-graecum *accessions were selected in order to represent most of the countries where it is grown. In case of *T. caerulea *all the nine accessions available at Saskatoon and Pullman were used in present study.

### Analysis of polymorphism detected in *T. foenum-graecum *and *T. caerulea*

Polymorphism in a given population is often due to existence of genetic variants represented by the number of alleles at a locus and their frequency of distribution in a population. Heterozygosity corresponds to a probability that two alleles taken at random from a population can be distinguished using the marker in question. Thus a convenient quantitative estimate of marker utility and the polymorphism detected can be given in terms of the mean heterozygosity and the marker index [18]. In *T. foenum-graecum *as well as in *T. caerulea *the H_av _and the marker index (MI) values for ISSR and RAPD markers [Table 3 and 4], respectively, did not differ significantly indicating that similar levels of polymorphism were detected by both the marker systems in the given germplasm pools. This was also confirmed by the high correlation co-efficent for ISSR and RAPD marker systems obtained for *T. foenum-graecum *and *T. caerulea *[Fig. 3 and 4]. Genetic diversity as measured by the heterozygosity was higher in *T. caerulea *(0.33) as compared to *T. foenum-graecum *(0.21). Based on allozyme diversity, the estimated mean heterozygosity values have been reported for self-pollinating species, *Vigna unguiculata*, H_av _= 0.027 [19] and *Vicia tetrasperma*, H_av _= 0.342 [20]. The heterozygosity value for *Vigna unguiculata *was lower while that for *Vicia tetrasperma *was higher as compared to *T. foenum-graecum *and *T. caerulea*. Based on ISSR markers, the estimates of genetic diversity, H_av _= 0.358 reported in cultivated pawpaw (*Asimia triloba*), was higher as compared to *T. foenum-graecum *and *T. caerulea *[13].

### Estimation of genetic relatedness in *T. foenum-graecum *and *T. caerulea*

Data collected with ISSR and RAPD marker systems were used to compare genetic similarity between various accessions of *T. foenum-graecum *and *T. caerulea*. The accessions could be a mixture of different genotypes. Therefore, to have a complete representation of a specific accession, DNAs from fifteen plants were mixed in equal proportion. Thus within accession diversity was eliminated and a complete banding profile of the accession was used for the analysis.

In *T. foenum-graecum*, ISSR and RAPD could detect almost similar level of polymorphism (72% with ISSR and 70.12% with RAPD). In the UPGMA analysis, *T. foenum-graecum *accessions from one country and the nearby region grouped together in some cases while they were placed in different clusters in certain cases. Accessions from Pakistan and Afghanistan grouped together in one cluster while accessions from India and Nepal grouped in another cluster. Moreover, the three accessions from Turkey fell in different clusters inspite of being geographically very close to each other. Thus, there was no clear clustering pattern of geographically closer accessions in the present study indicating that the association between genetic similarity and geographical distance was less significant. However, it is necessary to use more number of accessions from each geographical location to confirm the available pattern.

In *T. caerulea *also, ISSR (93.64%) and RAPDs (94.83%) detected almost equal level of polymorphism. *T. caerulea *showed more polymorphism as compared to *T. foenum-graecum*. In case of *T. caerulea *also, the UPGMA analysis showed that plants from different geographical regions were distributed in different groups. Here again, the accessions from Turkey were not grouped together. Two accessions from Turkey out grouped from the main cluster while one was grouped in the first cluster with Australia. In *T. caerulea *we could obtain three accessions from Turkey and only one accession from other countries. Therefore, it would be premature to comment precisely about the correlation of geographic distance and genetic diversity in this case. To confirm the available pattern it is necessary to use more number of accessions from each geographical location.

### Center of Origin and /or Center of Diversity for *Trigonella*

The place of origin of a species as explained by Vavilov is an area that contains a large amount of genetic variability of that species. According to him variation is a function of time, hence the region containing the greatest variation in a species would have supported and sustained that species for a longer time than the other regions suggesting that region to be the Center of Origin and/or Diversity. He set up eight geographic centers, two of which namely the Near Eastern and Mediterranean Centers extent into Turkey [21].

It has been postulated by Vavilov that the Near East region extending from Israel through Syria, Southern Turkey into Iran and Iraq and the Mediterranean Center including Spain, Morocco and Turkey is the Center of Origin of *Trigonella*, *Trifolium *and *Medicago *species [22]. In the present study both, *T. foenum-graecum *and *T. caerulea *accessions from Turkey exhibited more diversity. These results support Vavilov's hypothesis. The Indian accessions of *T. foenum-graecum *i.e. accession number 8686 (Khandwa), 8685(Mumbai), and 8687 (Patiala) separated by an aerial distance of 52 km, 104 km, and 135 km, respectively from each other, were genetically more similar (similarity index 0.893) and clustered together [Fig. 1]. However, the accessions of *T. foenum-graecum *from Turkey i.e. accession number 8692 (Malatya) and 8691 (Elbistan) separated by a distance of 100 km (similarity index value 0.875-0.745) were out grouped and were genetically more distant from each other although morphologically they were similar to each other as well to the accessions from other countries.

Turkey is one of the significant and unique countries in the world from the point of view of plant genetic resources and plant diversity. The country has more than 3,000 endemic plants and immense diversity has been reported in many legumes such as *Vicia*, *Medicago*, *Trifolium*, *Lathyrus*, *Onbrychis*, *Trigonella*, *Pisum*, *Cicer*, *Lens*, etc [23]. Many genera of cultivated plants like *Cicer*, *Lens*, *Pisum*, *Amygladus*, *Prunus*, *Triticum*, etc have their Center of Origin and/or Diversity in this country [22]. Vavilov designated southeastern Turkey and the adjoining Syria as the primary Center of Origin (now the center of diversity) for chickpea [24]. Similar to chickpea (and other grain legumes also), in *T. foenum-graecum *the large seeded cultivars are abounded around the Mediterranean region whereas the small seeded cultivars are predominated eastwards. Thus, Turkey may also be the primary Center of Origin of *T. foenum-graecum *and *T. caerulea*. However, this hypothesis needs to be confirmed by considering more accessions distributed over a wide geographic range especially from the Near East and the Central Mediterranean region.

## Conclusions

In conclusion, molecular markers allowed us to estimate the overall genetic diversity in *T. foenum-graecum *and *T. caerulea *and simultaneously revealed molecular based genetic relationship. In the UPGMA analysis, no significant correlation was observed between geographic distance and genetic diversity. Our data further supported the hypothesis of the Near East and the Central Mediterranean to be the Center of Origin and/or Diversity for *Trigonella *as put forth by Vavilov.

## Methods

### Plant material and DNA extraction

Seeds of *T. foenum-graecum *accessions were obtained from Plant Gene Resources of Canada (PGRC), Saskatoon, Canada. These accessions along with the TMP numbers and the country from where they have been collected are outlined in Table 1. Seeds for various *T. caerulea *accessions were obtained from PGRC, Saskatoon, Canada and USDA-ARS Plant Introduction Station at Pullman, Washington (W-6), and are detailed in Table 2. Fifteen plants of each accession were grown in pots for DNA isolation. Two gram of young leaf tissue was harvested for each plant and frozen in liquid nitrogen for DNA extraction. Plant DNA was extracted by the Doyle and Doyle method [25] and equal amount of DNA from each of the fifteen plants was pooled together for each accession.

### PCR amplification

#### ISSR

A set of 100 anchored micro satellite primers was procured from University of British Columbia, Canada. PCR amplification of 20 ng of DNA was performed in 10 mM Tris-HCI pH 7.5, 50 mM KCI, 1.5 mM MgCl2, 0.5 mM spermidine, 2% formamide, 0.1 mM dNTPs, 0.3 uM primer and 0.8 U of Taq DNA polymerase (Ampli-Taq DNA polymerase, Perkin Elemer, USA) in a 25 ul reaction using Perkin Elmer 9700 thermocycler. After initial denaturation at 94°C for 5 minutes, each cycle consisted of 30 seconds denaturation at 94°C, 45 seconds of annealing at 50°C, 2 minutes extension at 72°C along with 5 minutes extension at 72°C at the end of 45 cycles.

#### RAPD

RAPD analysis was performed using arbitary decamer primers procured from University of British Columbia, Canada. The reaction mixture (25 ul) contained 10 mM Tris-HCI pH 7.5, 50 mM KCI, 1.5 mM MgCl2, 0.5 mM spermidine, 0.1 mM dNTPs, 15 pmoles of primer, 20 ng genomic DNA, and 0.8 U of Taq DNA polymerase (Ampli-Taq DNA polymerase, Perkin Elmer, USA). Amplification was carried out using Perkin Elmer 9700 thermocycle for 40 cycles, each consisting of a denaturing step of 1 minute at 94°C, followed by annealing step of 1 minute at 36°C and an extension step of 2 minutes at 72°C. The last cycle was followed by 5 minutes of extension at 72°C. Amplified products were electrophoresed on 2% agarose gel using 0.5× TAE buffer (10 mM Tris HCl and 1 mM EDTA pH. 8.0) and visualized by ethidium bromide staining. The patterns were photographed and stored as digital pictures in gel documentation system. The reproducibility of the amplification was confirmed by repeating each experiment three times.

### Agarose gel electrophoresis 

Amplified products were electrophoresed on 2% agarose gel using 0.5x TAE buffer (10mM Tris HCl and 1mM EDTA pH. 8.0) and visualized by ethidium bromide staining. The patterns were photographed and stored as digital pictures in gel documentation system. The reproducibility of the amplification was confirmed by repeating each experiment three times.

#### Data analysis 

Unequivocally reproducible bands were scored and entered into a binary character matrix (1 for presence and 0 for absence). The genetic similarity among accessions was determined by Nei’s genetic distance [26]. A dendrogram was constructed based on the matrix of distance using Unweighted Pair Group Method with Arithmetic averages. Scores entered in matrix were analyzed using TAXAN version 4.0 software based on the degree of bands sharing. Similarity matrix was generated using Dice coefficient as SI = 2Nab/Na+Nb where Na = total number of bands present in lane a, Nb = total of bands in lane b, Nab number of bands common to lanes a and b [27]. The dice values were then used to perform the UPGMA analysis. To evaluate the robustness of the grouping formed the binary data matrix was subjected to bootstrapping using WinBoot program [28]. The phenogram was reconstructed 1000 times by repeating sampling with replacement and the frequency with which the groups were formed was used to indicate the strength of the group. Correlation co-efficient for the similarity matrices generated by ISSR and RAPD data in both, T.foenum-graecum and T.caerulea, were calculated by method of Smouse et al [29].   The expected heterozygosity, Hn for a genetic marker was calculated as: Hn = 1 - pi2, where pi is the allele frequency of the ith allele [26]. The arithmetic mean heterozygosity Hav for each marker class was calculated as Hav = Hn/n, where n = number of markers or loci analyzed [18]. The average heterozygosity for polymorphic marker (Hav)p was further derived as; (Hav)p = Hn/np; where np is the no. of polymorphic markers or loci [18]. Marker index (Ml) for each marker system was also calculated as, MI = E (Hav)p; where E = effective multiplex ratio [E = nβ where β is the fraction of polymorphic marker or loci,18].

## Authors' contributions

RD carried out the PCR and molecular genetic study, participated in the design of the study and performed statistical analysis. ML participated in procuring the seeds from different sources and monitoring experiments and result. LC a taxonomist participated in procuring, growing and analyzing the seeds obtained from different sources. PR and VG conceived of the study, participated in its design and co-ordination and monitored the experiments and the results.

All authors read and approved the final manuscript.

## Figures and Tables

**Table 1:** List of *T. foenum-graecum *accessions used in the present study

**Sr. No.**	**TMP. No.**	**Country**
1	8707	Afghanistan
2	1065	Canada
3	8698	Egypt
4	8696	Ethiopia
5	8686	India
6	8689	India
7	8687	India
8	8685	India
9	8675	Iran
10	8706	Nepal
11	8717	Pakistan
12	8718	Pakistan
13	8690	Turkey
14	8691	Turkey
15	8692	Turkey
16	8679	Egypt
17	8714	Yemen

**Table 2:** List of *T. caerulea *accessions used in present study

**Sr. No.**	**Source**	**TMP\PI No.**	**Country**
1	Saskatoon	TMP = 8704	Turkey
2	Saskatoon	TMP = 8738	Unknown
3	USDA	PI = 345743	U.S
4	USDA	PI = 244288	Spain
5	Saskatoon	TMP = 8750	Unknown
6	USDA	PI = 206486	Turkey
7	USDA	PI = 186283	Australia
8	USDA	PI = 206901	Turkey
9	Saskatoon	TMP = 8752	Unknown

**Table 3:** Comparison of DNA marker systems in *T. foenum-graecum*

**Marker System**	**No. of primers**	**% Polymorphism**	**Average No. Of bands/Primer**	**H_av_**	**(H_av_)p**	**MI**
ISSR	14	72	7.14	0.2145	0.297	1.53
RAPD	22	70.12	7.45	0.203	0.289	1.51
ISSR + RAPD	36	70.8	7.33	0.211	0.298	-

**Table 4:** Comparison of DNA marker systems in *T. caerulea*

**Marker System**	**No. Of primers**	**% Polymorphism**	**Average No. Of bands/Primer**	**H_av_**	**(H_av_)p**	**MI**
ISSR	18	93.64	9.61	0.330	0.354	3.17
RAPD	10	95.83	9.7	0.346	0.361	3.35
ISSR + RAPD	28	94.05	9.65	0.338	0.359	-
